# Fragile temporal prediction in patients with schizophrenia is related to minimal self disorders

**DOI:** 10.1038/s41598-017-07987-y

**Published:** 2017-08-15

**Authors:** Brice Martin, Nicolas Franck, Michel Cermolacce, Agnès Falco, Anabel Benair, Estelle Etienne, Sébastien Weibel, Jennifer T. Coull, Anne Giersch

**Affiliations:** 1 Centre Ressource de Réhabilitation psychosociale et de remédiation cognitive, Centre Référent Lyonnais en Réhabilitation et en Remédiation cognitive (CL3R) Hôpital du Vinatier; CNRS UMR 5229, Lyon, France; 2 INSERM U1114, Pôle de Psychiatrie, Fédération de Médecine Translationnelle de Strasbourg (FMTS), Centre Hospitalier Régional Universitaire of Strasbourg, Université de Strasbourg, Strasbourg, France; 3 Service Universitaire de Psychiatrie, Centre Hospitalier Universitaire Ste Marguerite, 13009 Marseille, France; 4 Laboratoire des Neurosciences Cognitives (UMR 7291), Aix-Marseille Université & CNRS, 3 Place Victor Hugo, 13331 Marseille cedex 3, France

## Abstract

Patients with schizophrenia have difficulty in making sensory predictions, in the time domain, which have been proposed to be related to self-disorders. However experimental evidence is lacking. We examined both voluntary and automatic forms of temporal prediction in 28 patients and 24 matched controls. A visual cue predicted (temporal cue) or not (neutral cue) the time (400 ms/1000 ms) at which a subsequent target was presented. In both patients and controls, RTs were faster for targets presented after long versus short intervals due to the temporal predictability inherent in the elapse of time (“hazard function”). This RT benefit was correlated with scores on the EASE scale, which measures disorders of the self: patients with a high ‘self-awareness and presence’ score did not show any significant benefit of the hazard function, whereas this ability was preserved in patients with a low score. Moreover, all patients were abnormally sensitive to the presence of “catch” trials (unexpected absence of a target) within a testing block, with RTs actually becoming slower at long versus short intervals. These results indicate fragility in patients’ ability to continuously extract temporally predictive information from the elapsing interval. This deficit might contribute to perturbations of the minimal self in patients.

## Introduction

Predicting events in time is inherent to our mental life, and may play a role in the temporal structure of consciousness. Indeed, it has been proposed that predictions help to bridge isolated events together and to build the sense of stability and continuity that constitutes our subjective life^1^. Its disruption in patients with schizophrenia^2–4^ might be involved in minimal self disorders^5, 6^, which are core disorders affecting the inner experience of patients. Self-disorders can already be observed during the prodromal phase of schizophrenia, and are possibly related to all cardinal symptoms of schizophrenia^7^. To test the link between time and minimal self disorders, we explored patients’ ability to benefit from sensory expectations, together with an evaluation of minimal self disorders by means of the EASE scale^8^.

Having an expectation that a sensory signal will arrive at a particular moment in time helps us to adapt behavior to external events. This process, however, may be impaired in patients. Indeed, it has been found to be impaired under the influence of ketamine^9^, a psychotomimetic drug that mimics the symptoms of the prodromal phase of schizophrenia, including both positive and negative symptoms as well as cognitive impairments^10, 11^. Our aim is to measure to what extent this is also true in schizophrenia.

## Prediction disturbances in schizophrenia

Many results in the literature suggest that patients with schizophrenia are impaired at making predictions. Some of the first studies were in the domain of motor control. They suggested that the processes involved in predicting the sensory outcome of an action and comparing it to the real outcome are impaired, leading to agency disorders^12–16^. Impaired predictions may also be related to mismatch negativity disturbances in patients. Mismatch negativity is explored by displaying repetitive stimuli interspersed with oddballs. EEG recordings taken during the presentation of such stimuli reveal evoked potentials to the oddballs, called “mismatch negativities”, whose amplitude is reduced in patients^17–21^. Aberrant predictions^22, 23^ may underlie patients’ difficulty in distinguishing between oddball versus repeated stimuli^20, 24, 25^. Recently it has been proposed that prediction impairments in patients may concern the prediction of time in particular^26, 27^. The existence of an elementary form of temporal prediction impairment in patients with schizophrenia has been proposed at the millisecond level on the basis of automatic responses in simultaneity judgments, as evaluated with the Simon effect^28, 29^. It was shown that after the display of two consecutive stimuli separated by a short stimulus onset asynchrony of less than 20 ms, healthy volunteers automatically moved their attention towards the second stimulus^30^. These results indicated that the two stimuli were automatically distinguished in time, even though they were judged to be simultaneous. Logically, this means that the two stimuli were processed successively. Since stimulus processing takes time^31^, processing of the first stimulus entails the risk of missing the second one. However, in a task composed almost entirely of trials containing two consecutive stimuli (stimuli were presented simultaneously only very rarely), this risk can be minimised by predicting in advance that the stimulus display will contain a sequence of two discrete events. While attention moved successively from one stimulus to another in healthy controls, patients with schizophrenia remained focused on the first stimulus. This was interpreted as an impairment in temporal prediction at the millisecond scale.

This impairment at the millisecond level might reflect a basic difficulty in connecting the flow of events^32^, thereby impeding the ability to encode the passage of time at even longer time-scales. This could account for patients’ variability in making explicit judgements of duration (e.g. is stimulus A shorter/longer than stimulus B?) in the range of hundreds of milliseconds to seconds^33^. To further explore the link between patients’ ability to process duration in this time-range and their ability to make temporal predictions, we measured their performance on a cued version of the “variable foreperiod” reaction time task, in which cues predicted or not when a target would appear^34^.

## Variable foreperiod paradigm

The variable foreperiod paradigm can be used to index implicit temporal preparation, which is akin to what happens when waiting for a red traffic light to turn green. At a variable interval (or “foreperiod”) after an initial fixation point, a target is displayed on the screen and participants have to detect its appearance by pressing a response key as quickly as possible. As the duration of the interval increases, reaction times (RT) to detect the target get faster^35–38^. The speeding of RTs at longer intervals is known as the “variable foreperiod effect” and reflects the increasing sense of expectancy that a target will occur as time elapses, leading to an ever-heightening sense of temporal certainty. This increasing probability over time is termed the “hazard function” and can be used to generate and update temporal predictions dynamically.

This form of temporal processing is mainly subconscious and unintentional^38^. It is distinct from mechanisms that allow us to voluntarily anticipate an event precisely in time. Voluntary temporal prediction has been measured with the “temporal orienting” task, in which temporal cues indicate the time interval between the initial fixation point and the target^34, 39^. Temporal cues allow attentional resources to be oriented to specific moments in time, leading to faster RTs for targets appearing at the times predicted by the cue. In neutral cue conditions, in which the cue provides no temporal information, RTs are faster at long than short delays, due to the influence of the hazard function. In temporal cue conditions, by contrast, reaction times are equally fast at short and long delays^40^. The benefit of temporal versus neutral cues is therefore primarily seen at short delays^41^ since RTs at long delays are fast anyway, due to the influence of the hazard function. fMRI studies show the effects of temporal cues and the hazard function to be sub-tended by different, though partially overlapping, neural networks^42^ confirming the functional distinction between the implicit use of the hazard function to make temporal predictions versus the voluntary use of temporal cues.

Beside temporal cues, the effects of the hazard function can also be tempered by the probability of target occurrence. Indeed, Drazin^43^ noted that including a proportion of trials in which the target was not presented (catch trials) modulated the effects of the hazard function on RT. Increasing the proportion of catch trials significantly slowed RTs at long delays, essentially flattening out the RT slope that is normally seen from short to long delays^40, 43, 44^. Näätänen^44^ suggested that when catch trials are introduced into a block of trials, the subjective probability of target expectancy actually *decreases* as the delay lengthens, thereby impairing participants’ normal level of preparation. Correa *et al*.^40^ termed this a ‘dispreparation’ effect.

## Variable foreperiod effects and schizophrenia

The variable foreperiod task has been employed in experimental studies of schizophrenia for several decades^45–49^. These studies indicate a relative preservation of the influence of the hazard function on RTs in schizophrenia, though reveal abnormal performance when foreperiods are fixed rather than variable^45–47^. However the effects of the hazard function in patients were investigated only over long time scales (longer than one second), and no study has yet investigated the impact of temporal cues on the influence of the hazard function in schizophrenia. It therefore remains possible that patients are selectively impaired at orienting attention to fixed moments in time, especially since patients are known to have attentional difficulties^50, 51^. The temporal cue condition of the temporal orienting paradigm allows us to explore this hypothesis. In addition, given patients’ difficulty in adapting to changing contexts^22, 23, 52^ we also measured the effect of varying the proportion of catch trials on patients’ performance in this paradigm. Finally, we investigated the relationship between putative difficulties in temporal prediction in patients with minimal self disorders, e.g. the most basic level of the self, which is one of the most documented levels of self disturbance in schizophrenia^7, 8, 53^. Gallagher^54^ defines the minimal self as “a consciousness of oneself as an immediate subject of experience” (p. 15) and as “the pre-reflexive point of origin for action, experience and thought”. An important aspect of minimal self is the sense of self-awareness and presence, i.e. the feeling that we are immersed in the world, continuously following the flow of events. This sense of self-awareness and presence has been described as being perturbed in patients, and might be related to impairments in temporal prediction.

Therefore, by means of the temporal orienting paradigm, we explored patients’ ability to predict time, over intervals of 400 to 1000 ms, and its relation to minimal self disorders. In view of the hypothesis that temporal prediction is impaired in patients with schizophrenia, and that this is related to minimal self disorders, we expected the beneficial effects of the hazard function or temporal cues to be smaller in patients than controls, at least in those displaying symptoms related to minimal self disorders. Self disorders were explored by means of the EASE (Examination of Anomalous Self-Experience) scale, which evaluates different aspects of the minimal self, such as cognition and stream of consciousness, self-awareness and presence, or bodily experiences (a complete list and description can be found in the Methods section). We focused on the ‘self-awareness and presence’ sub-scale, which is based on items that explore the most basic self disorders, by looking for symptoms related to a decreased or distorted feeling of being present in the here and now, difficulty in understanding what is usually taken as a given and is obvious (‘a loss of natural evidence’), and the perplexity and anxiety that accompanies this difficulty. Finally, given patients’ difficulty in adapting to changing contexts^22, 23, 52^, we hypothesized that patients would diverge from controls more when targets were not systematically presented on every trial (i.e. in the presence of catch trials).

## Results

We report the main results here. Complementary statistics, with effect sizes, can be found in the Supplementary Material.

### Effect of catch trials on reaction time

There was a significant interaction between foreperiod (400 ms/1000 ms) and catch-trial percentage (0%/25%) (F[1, 48] = 62.4, p < 0.001, partial η^2^ = 0.57). In the condition without catch trials (target absent in 0% of trials), the post-hoc analysis showed that RTs decreased significantly from the 400 ms foreperiod to the 1000 ms foreperiod (by 13 ms, p < 0.001). Speeding of RTs at long foreperiods indexes the influence of the hazard function, and is evident in the absence of catch trials. In the condition with catch trials (target absent in 25% of trials), RTs increased (by 6 ms) from the 400 ms foreperiod to the 1000 ms foreperiod (p < 0.005).

### Between-group differences

**Figure 1:**
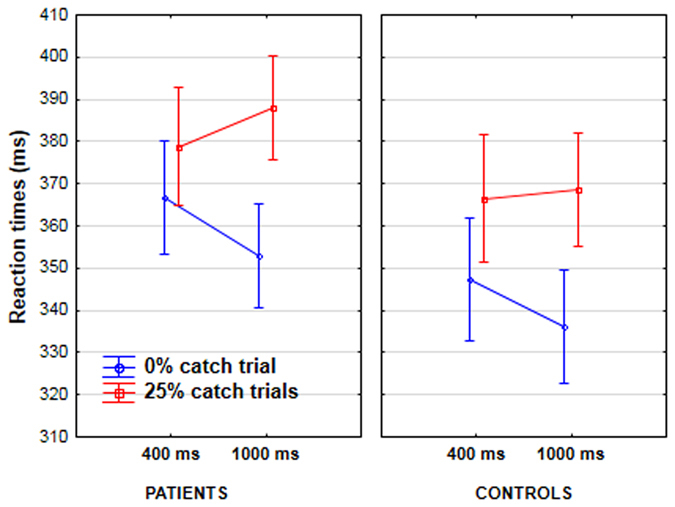
Reaction times in patients (lefthand graph) and controls (righthand graph) as a function of target condition (0% catch trial in blue, vs. 25% catch trials in red), and the foreperiod between the cue and the target (400 vs. 1000 ms).

There was a significant interaction between foreperiod, catch-trial percentage and group (F[1, 48] = 4.52, p < 0.05, partial η^2^ = 0.09). There was no other interaction with group. Notably, the nature of the cue did not interact with group (see below) and so the following results are averaged over the two cue conditions. The post hoc-analysis showed that in the condition without catch trials, RTs decreased significantly from the 400 ms foreperiod to the 1000 ms foreperiod, in both groups (by 14 ms in patients, p < 0.001, and by 11 ms in controls, p < 0.001). By contrast, in the condition with catch trials (target absent in 25% of trials), the RTs did not change from the 400 ms foreperiod to the 1000 ms foreperiod in the control group (there was a difference of only 2 ms between the two intervals, p > 0.9), but increased significantly in patients from the 400 to 1000 ms foreperiod (by 10 ms, p < 0.005) (Fig. 1). We also explored the phasic effects of an absent target by comparing RTs for trials that immediately followed catch trials versus trials that immediately followed a target-present trial. Results showed a significant interaction between this variable and foreperiod (F[1, 48] = 15.2, p < 0.001, partial η^2^ = 0.24). RTs increased by 19 ms from 400 to 1000 ms after a catch trial (p < 0.001 in the Tukey post-hoc analysis) whereas they remained stable after a target-present trial (4 ms of difference, *n*.*s*.). This effect did not differ significantly between groups.

### Effect of temporal cues on reaction time

**Figure 2:**
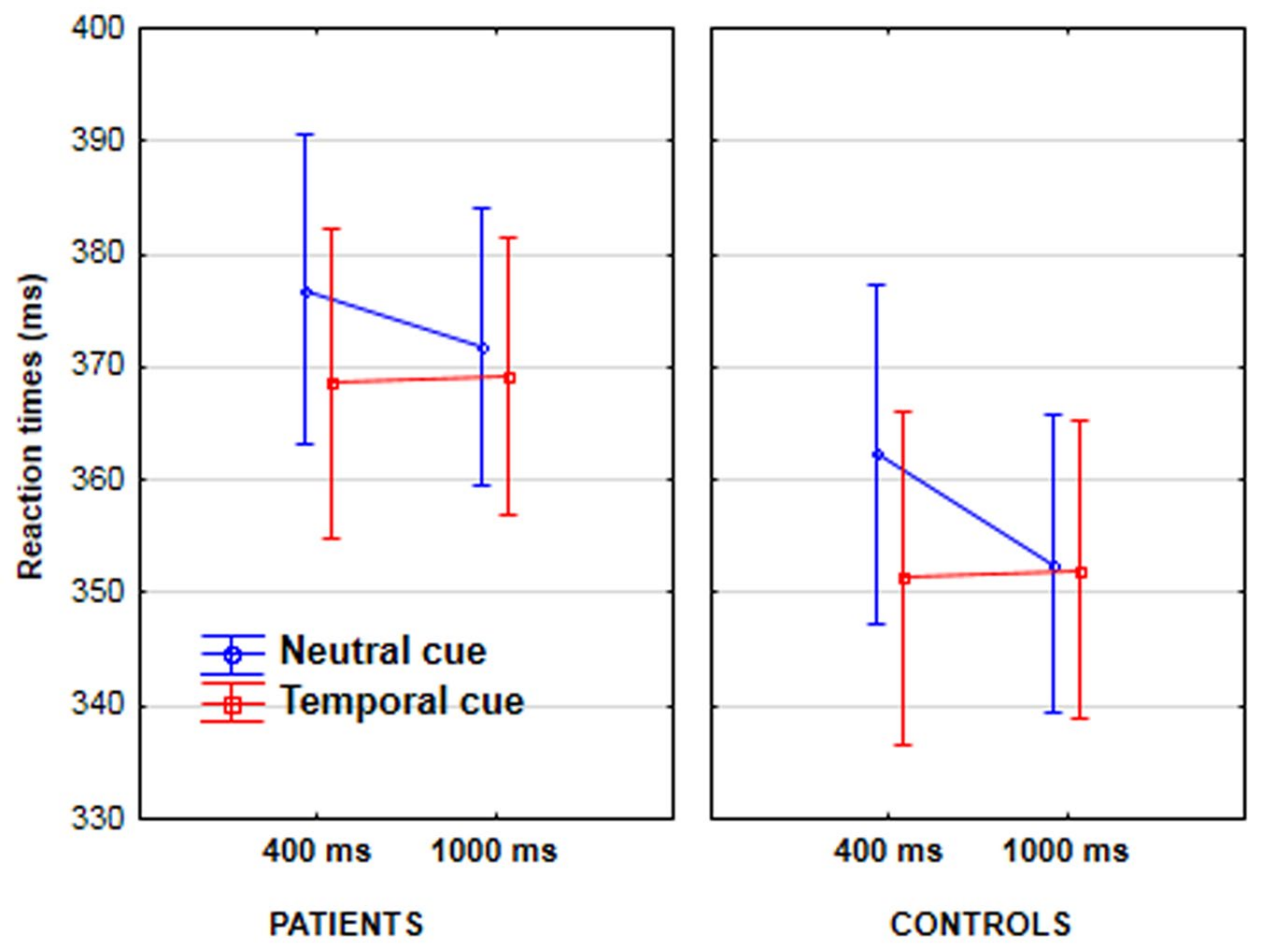
Reaction times in patients (lefthand graph) and controls (righthand graph) as a function of the presence or absence of temporal cues (neutral cues in blue, vs. temporal cues in red), and the foreperiod between the cue and the target (400 vs. 1000 ms).

There was a significant interaction between foreperiod (400 ms/1000 ms) and cue type (temporal/neutral) (F[1, 48] = 10.6, p < 0.005, partial η^2^ = 0.18), but no interaction with group (F < 1). To ensure that patients benefitted from temporal cues as well as controls, we measured the cue effect in both groups separately (Fig. 2). The interaction between foreperiod and cue type was significant in the control group (F[1, 22] = 8, p < 0.01, partial η^2^ = 0.27) with post-hoc Tukey analysis showing that RTs were faster by 11 ms at the 400 ms foreperiod in the presence of temporal versus neutral cues (p < 0.005). In the patient group, the interaction between foreperiod and cue type tended towards significance (F[1, 26] = 2.9, p = 0.098, partial η^2^ = 0.1). The Tukey post-hoc analysis showed that the pattern of RTs was similar to controls, i.e. faster RTs at 400 ms in the presence of temporal versus neutral cues (by 8 ms, p < 0.01).

### Correlation with neurocognitive and clinical measures

We were interested in how minimal self disorders relate to the implicit sense that the target is increasingly likely to occur as time elapses (the effect of the hazard function). This effect is contaminated by voluntary attentional mechanisms in the temporal cue condition. We therefore correlated clinical ratings with the profile of RT performance in the neutral condition. The decrease in RT from short to long foreperiods in the neutral condition varied between patients, even in the absence of catch trials. To explore whether this was related to patients’ clinical symptoms we calculated correlations between clinical or neurocognitive measures and the magnitude of the slope between short and long foreperiods in the 0% and 25% catch trials conditions. Magnitude was calculated by subtracting reaction times in the neutral condition at long foreperiods from those at short foreperiods. There was no correlation between PANSS scores and the magnitude of the RT slope in either the 0% or 25% catch trial condition. EASE scores of self-awareness and presence correlated with the RT slope in the 0% catch trials condition (N = 23, r = −0.4, p < 0.05): patients who were less immersed in the world (highest self-awareness and presence scores) displayed a shallower RT slope from the 400 ms to 1000 ms foreperiod (Fig. 2). The full set of correlations can be found in the supplementary material.

**Figure 3:**
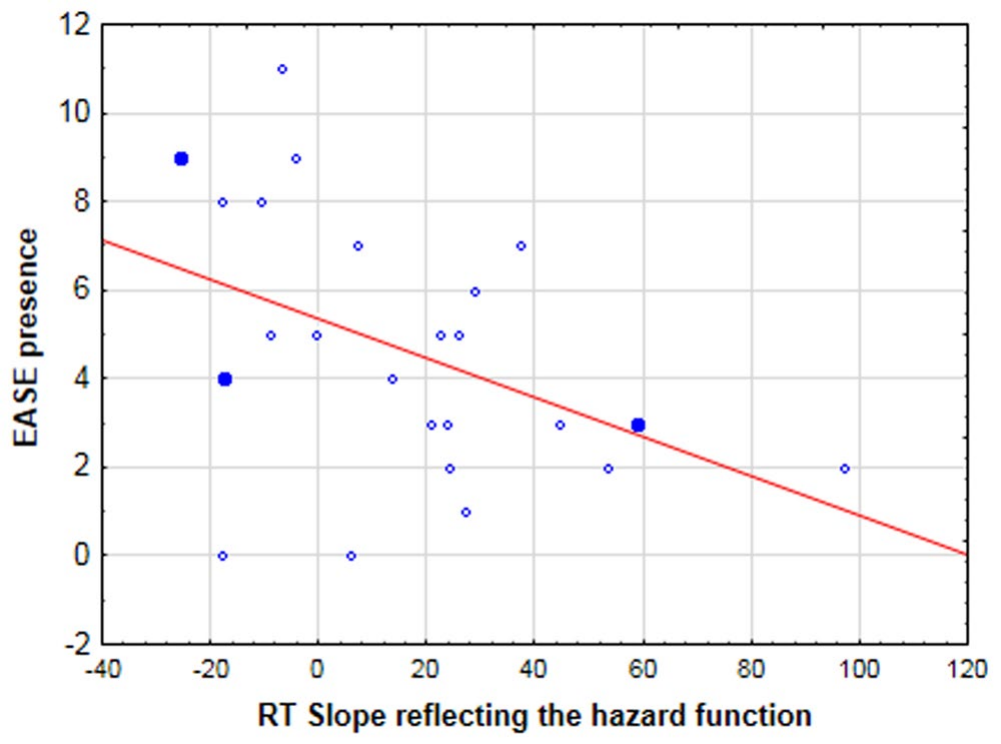
Scatterplot illustrating the correlation between the EASE score of ‘self-awareness and presence’ and the RT effects of the hazard function in the 0% catch trial condition. A higher ‘self-awareness and presence’ score corresponds to more severe symptoms. A lower RT slope score reflects a smaller (or even absent) benefit of the hazard function.

We also looked for correlations between clinical measures and patients’ response to a lack of target in the previous trial (i.e. for catch trial blocks, we calculated the RT difference between 400 and 1000 ms for trials following a catch trial vs. those following a target-present trial. Since this response is independent of whether the cue is temporal or neutral, data were averaged over cue conditions). EASE scores of self-awareness and presence correlated with the change in RT slope for trials that followed a catch trial vs. those that followed a target-present trial (N = 23, r = 0.6, p < 0.005). Patients with the highest self-awareness and presence scores were those for whom the RT difference between 400 ms and 1000 ms did not vary according to the preceding trial: RTs increased between 400 and 1000 ms systematically for all trial types. In contrast, for patients with the lowest self-awareness and presence scores the magnitude of the RT slope differed according to the nature of the preceding trial: RTs increased between 400 and 1000 ms mainly after a catch trial (Fig. 3).

Subgroup analyses of patients were conducted on the basis of EASE scores and can be found in the supplementary material.

## Discussion

Our results show that when a target is displayed either 400 or 1000 ms after a neutral warning cue, response times are faster for targets appearing after long, rather than short, intervals (“foreperiods”). This effect is observed when participants do not know in advance when the target will appear, but are sure that a target is bound to appear (i.e. during blocks with no catch trials). The effect is attenuated however, when a temporal cue precisely indicates the duration of the forthcoming foreperiod. In this case, participants are equally fast at both short and long foreperiods. These effects replicate results described in the literature^37, 40, 42, 55^, and reflect the increasing expectancy of target appearance as time elapses (the “hazard function”) in neutral cue trials and the ability to voluntarily attend to a precise moment in time in temporal cue trials. Although the benefit in RTs is relatively small for both effects (<20 ms), it is highly reliable. Our data further reveal a normal benefit of the hazard function in the group of patients with schizophrenia, again in agreement with previous literature^45–49^. Nevertheless, we extend this literature first by showing that temporal preparation is unimpaired in patients at a relatively short timescale (below 1000 ms). Moreover, we show for the first time, that the ability to use symbolic temporal cues to predict precisely when a target will appear is preserved in patients. Taken as a whole, these data suggest patients had no difficulty in making temporal predictions. They also show that certain timing and attentional processes are preserved. This is consistent with the fact that patients often cope well in everyday life, which would otherwise be impossible if timing were generally impaired. However, when taking into account patients’ symptoms, and when examining the effect of catch trials, it seems that the influence of the hazard function is more fragile in patients than first appears.

Our results indicate that when the appearance of the target becomes uncertain (i.e. during blocks with 25% catch trials), patients’ RTs are actually slower at long versus short foreperiods. Crucially, this differs from results in controls, in which RTs were no different at short and long foreperiods in the 25% catch trial condition. The inclusion of catch trials is known to attenuate the speeding of RTs normally seen at long foreperiods, thereby flattening out the RT slope from short to long foreperiods^43^. Our control group data therefore perfectly illustrate the normal influence of catch trials on performance (sometimes referred to as “dispreparation”). By contrast, the inclusion of catch trials attenuated the influence of the hazard function to an even greater extent in patients: RTs were not just as slow at long foreperiods as at short ones, but were actually even slower.

Moreover, the correlation between EASE scores and the slope of RT performance in neutral trials indicates that the more patients show disturbances in the minimal self, the more the benefit of the hazard function is reduced, suggesting that the severity of the disease is associated with abnormalities in the implicit processing of time. These results are reminiscent of the effects of ketamine in healthy volunteers, which also significantly attenuated the behavioural benefit of the hazard function^9^. Interestingly, ketamine is considered as a model for first-episode, rather than chronic, psychosis^56^. Since minimal self disorders have mostly been observed in the early stages of schizophrenia^57–59^, our results fit well with these pharmacological findings. Moreover, the fact that correlations are found with the kinds of disorders that precede the emergence of cardinal symptoms, rather than with PANSS scores, also fits with the idea that elementary time prediction deficits are associated with core disorders in schizophrenia. Coull *et al*.^9^ also found that ketamine did not impair participants’ ability to voluntarily use temporal cues to predict the time of target onset, which is also true of the patients in our study. Just like controls, patients were able to voluntarily use temporally valid cues to predict the time of target onset and so speed performance. This result is also in line with recent work showing that patients with schizophrenia are able to use explicit cues to improve their performance^60–62^.

Our results indicate that not all predictive coding mechanisms are impaired in schizophrenia. However, dispreparation, observed in catch trial blocks, is affected. It might be argued that dispreparation is related to motivational problems in patients, who could become disengaged in the task due to the presence of catch trials. This is unlikely, though. First, we found no correlation between performance and negative symptoms. Second, a lack of motivation would have resulted in increased RTs at both 400 and 1000 ms delays. This was not the case. The dispreparation effect observed in the group of patients as a whole might instead be a combination of fragile temporal preparation (at least in patients with high self-awareness and presence scores) and an overreaction to the additional uncertainty induced by the absence of the target in a catch trial, which would perturb performance on the next trial (see supplementary data for additional analyses in favour of this interpretation). Catch trials represent a form of prediction error. Our results in the 0% catch trial condition show that patients are able to make use of the hazard function to predict when a target will occur. If, in the 25% catch trial condition, the target is not displayed, it will be recorded by patients as a prediction error. Patients might be oversensitive to such prediction errors, with the result that they simply stop preparing for the possibility that the target will appear at the long foreperiod, i.e. dispreparation. This interpretation fits with the idea that prediction error processing is aberrant in patients^22, 23^, and is not inconsistent with the widely reported reduction of the MMN in patients^21^. The MMN represents a heightened response to a rare event. In our experiment, results show that the occasional lack of a target discourages patients from predicting when a late stimulus will appear. This affects performance throughout the entire task for patients with high scores on the EASE self-awareness and presence sub-scale, but appears to affect performance particularly on trials that occur immediately after a catch trial in patients with low self-awareness and presence scores. It appears therefore that it is the uncertainty regarding whether or not a target will appear that impedes patients from forming temporal expectations about when the target will appear. Such expectations are also engaged in typical MMN tasks when rare events, such as a sensory modification to the target, are detected. What might be difficult to understand here is that our results seem to suggest that heightened sensitivity to prediction error leads ultimately to a reduced sense of expectation. We suggest this can be reconciled with the MMN literature by postulating that during an MMN task, or any given task, we naturally adapt to the statistical probability of the entire range of events occurring during the task, including rare events. If absent-targets are considered rare, healthy volunteers will therefore continue to have a minimum degree of expectation that a target will eventually arrive, even during blocks with catch trials. In more typical MMN tasks, this means that they will continue to expect the most frequent event. By contrast, patients appear to be unable to deal with such uncertainty. They are able to detect prediction errors (e.g. the absence of a target), but since the mechanisms involved in such predictions are resource-demanding they relinquish their state of expectation more easily than controls and so fail to benefit from the temporal predictability conveyed by the hazard function. Moreover, we have found a graded profile of effect. Patients with high scores on the EASE appear to give up throughout the entire task, whereas patients with low scores give up transiently only after a catch trial. Patients with low scores are able to re-mobilize expectation mechanisms, but will over-react once again whenever a catch trial is presented. This interpretation also fits nicely with the clinical observation that patients with schizophrenia are upset by unexpected and unusual sensory events^63^.

In sum, our results indicate that patients present a degree of fragility in their implicit expectation of events in time. This result is in line with recent studies investigating automatic temporal expectation mechanisms in the tens of milliseconds range in schizophrenia^28, 29, 32, 64^, and generalizes these results to durations in the hundreds of milliseconds range. As indicated by correlations between EASE scores and the hazard function effect, this disturbance could lead to minimal self disorders, especially disturbances of the sense of self-awareness and presence as postulated by psychiatric phenomenology^3, 4, 6, 65^. By losing the sense of time, patients also lose their sense of immersion or pre-reflexive tuning to their environment. Our data indicate that uncertainty regarding the very occurrence of a future event may aggravate this already fragile process.

## Methods

### Participants

Participants were 28 stabilized outpatients and 24 matched controls (Table 1).

**Table 1:** Demographic characteristics of each group of participants.

	Patients	Controls	Group effect
Gender M/F	23/5	18/6	Chi² < 1, n.s.
Age (mean+/−SD)	31,0 (+/−7,9)	30,6 (+/−8,4)	F < 1, n.s.
Years of education (mean+/−SD)	12, 6 (+/−1,9)	13,6 (+/−1,8)	F[1,50] = 3.6, n.s.
Medication (typical, atypical, no medication)	0/25/3		
Mean+/−SD dose of chlorpromazine equivalents / patient	289 mg/day (+/−252)		
PANSS positive symptoms (mean+/−SD)	15,2 (+/−5,5)		
PANSS negative symptoms (mean+/−SD)	20,8 (+/−8,2)		
PANSS general symptoms (mean+/−SD)	38,9 (+/−11,7)		
PANSS total (mean+/−SD)	74,1 (+/−22,3)		
EASE ‘Cognition and stream of consciousness’ (mean+/−SD) maximum = 17	6,6 (+/−3,9)		
EASE ‘Self-awareness and presence’ (mean+/−SD) maximum = 18	4,8 (+/−3)		
EASE ‘Bodily experiences’ (mean+/−SD) maximum = 9	1,6 (+/−1,5)		
EASE ‘Demarcation – transitivism’ (mean+/−SD) maximum = 5	1 (+/−0,9)		
EASE ‘Existential reorientation’ (mean+/−SD) maximum = 8	1,4 (+/−1,4)		
EASE total (mean+/−SD)- maximum = 57	15,4 (+/−7,7)		

The project was approved by a local ethics committee (CPP Sud Est VI), and informed written consent was obtained, before the study, from each patient and control participant. All methods have been conducted in accordance with the recommendation of the Declaration of Helsinki.

Psychiatric diagnoses were established by a senior psychiatrist from the department. Diagnoses fulfilled the Diagnostic and Statistical Manual of Mental Disorders, Fifth Edition, critera for a diagnosis of schizophrenia. Exclusion criteria for patients and controls were the intake of benzodiazepines, a history of alcohol and drug dependency, neurological and medical pathologies, a disabling sensory disorder, and general anesthesia in the 3 months prior to testing. An additional exclusion criterion for controls was psychotropic medication in the 3 weeks prior to testing. One subject was excluded from the analysis in each group due to missing data. Replacing the missing data by the data averaged over the group did not change the results.

In addition to typical clinical scales, the EASE scale was administered by the first author of the paper (MB), who was trained on its use. The EASE is a 57 item semi-structured interview designed to explore disorders of the minimal self ^8^.

Five domains were explored:

- Cognition and stream of consciousness (17 items). This domain explores disturbances in the stream of consciousness associated with the feeling of a gap between one’s own thoughts and the self, leading to the loss of “mineness” of mental experience.
- Self awareness and presence (18 items). This domain explores a broad range of phenomena that can be defined as a lack of immersion in the world.
- Bodily experiences (9 items). This domain explores a broad range of bodily experiences characterized by the feeling of being detached from oneself and one’s actions, as if in a third- person perspective or without any perspective at all.
- Demarcation – transitivism (5 items). This domain explores a range of experiences characterized by a difficulty discriminating self from not self ^8^.
- Existential reorientation (8 items). The patient manifests a fundamental reorientation with respect to his general metaphysical world view and/or hierarchy of values, projects and interests.

Each item was scored on a 2 point likert scale: 0 as absent or questionably present and 1 as definitely present. Each interview took 30–120 min, was video filmed and conducted by BM after training by one of the authors of the EASE scale (JP). The inter-rater reliability for the EASE score was examined on the basis of 30% of the video-taped interviews by MC, who was also trained by JP. The inter-rater reliability was found to be excellent with kappa = 0.9.

### Apparatus

The experiments were run on a Pentium 4 PC and programmed with E Prime2.0. Stimuli were displayed on a monitor with a refresh rate set to 60 Hz. Stimulus presentation occurred in an environment of low-intensity ambient light (0.1 cd/m^2^); daylight did not enter the room. The distance between the screen and the participants was held constant, at 100 cm, by means of a chinrest.

### Stimuli

**Figure 4:**
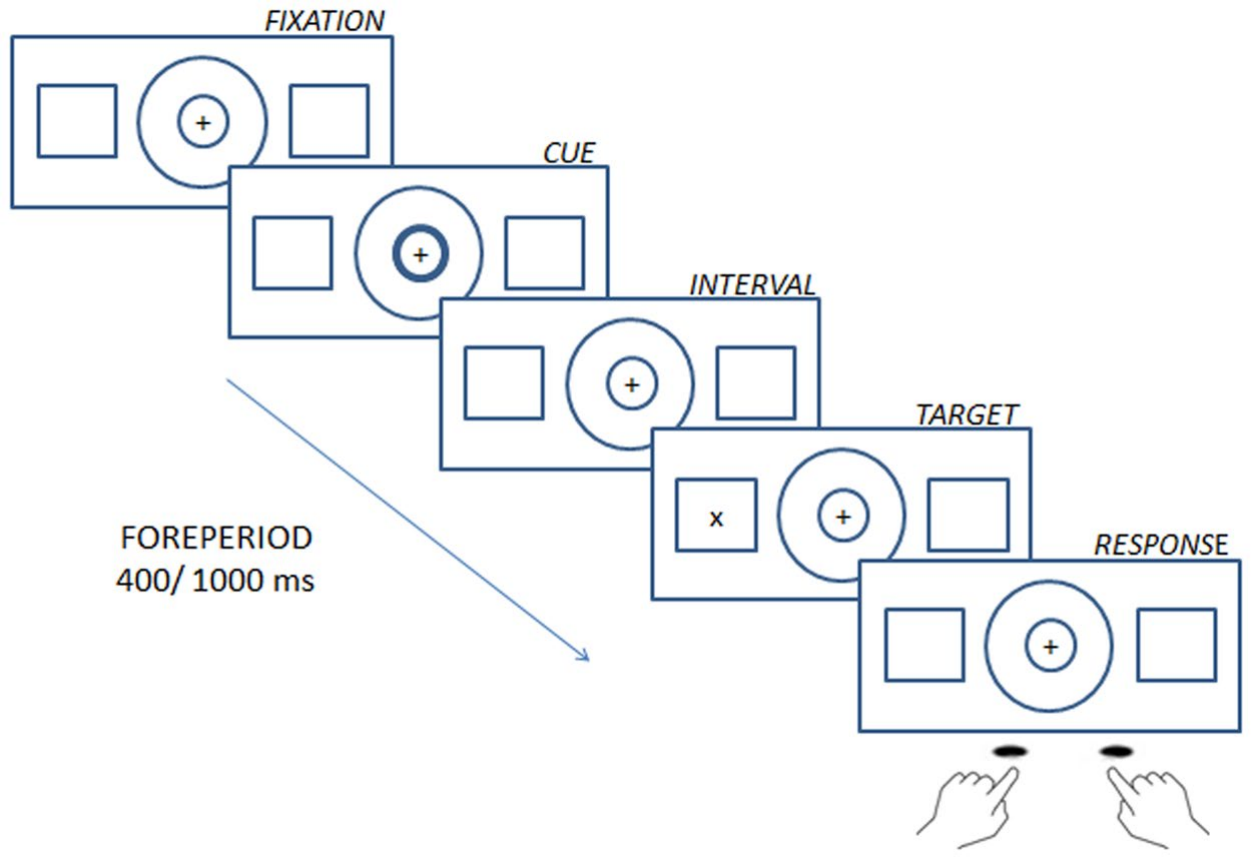
Sequence of events in a trial. Stimuli were presented in grey on a black background.

The background display consisted of a central fixation point (“+”) surrounded by two circles, and two peripheral boxes left and right of centre (Fig. 4). The trial began when one or both circles were briefly (100 ms) highlighted, representing the cue. Next, the screen remained blank for a variable delay of 400 or 1000 ms. The target was displayed in either the left or right box for 100 ms. If no response to the target was made, the next trial began after a randomized delay of 1600 or 2000 ms.

If the inner circle of the central cue was briefly highlighted (Fig. 4), it indicated that the target would appear after a short interval, or “foreperiod” (400 ms, short temporal cue). If the outer circle was highlighted, it indicated that the target would appear after a long foreperiod (1000 ms, long temporal cue). When the two circles were highlighted together, the target could appear after either a short or long interval (neutral cue). Therefore, temporal cues gave precise temporal information as to when the target would appear whereas the neutral cue provided no temporally precise information. The target was either the letter ‘X’ or the symbol ‘+’. The two targets appeared with a probability of 0.50, and were varied only to avoid boredom. The response was the same whatever the form of the target.

### Procedure

Patients and controls were instructed to respond to the target as quickly and accurately as possible and to use the temporal cue to anticipate the moment of target onset. Participants were instructed to press a right key when the target was presented on the right, and a left key when the target was presented on the left. This discrimination task was used to prevent anticipatory responding, since participants had to first process the side on which the target was displayed before pressing a response key.

The experiment consisted of one block of 64 practice trials, during which participants learnt the temporal association between the inner/outer circle component of the cue and the short/long interval, and eight blocks of 120 experimental trials.

**Figure 5:**
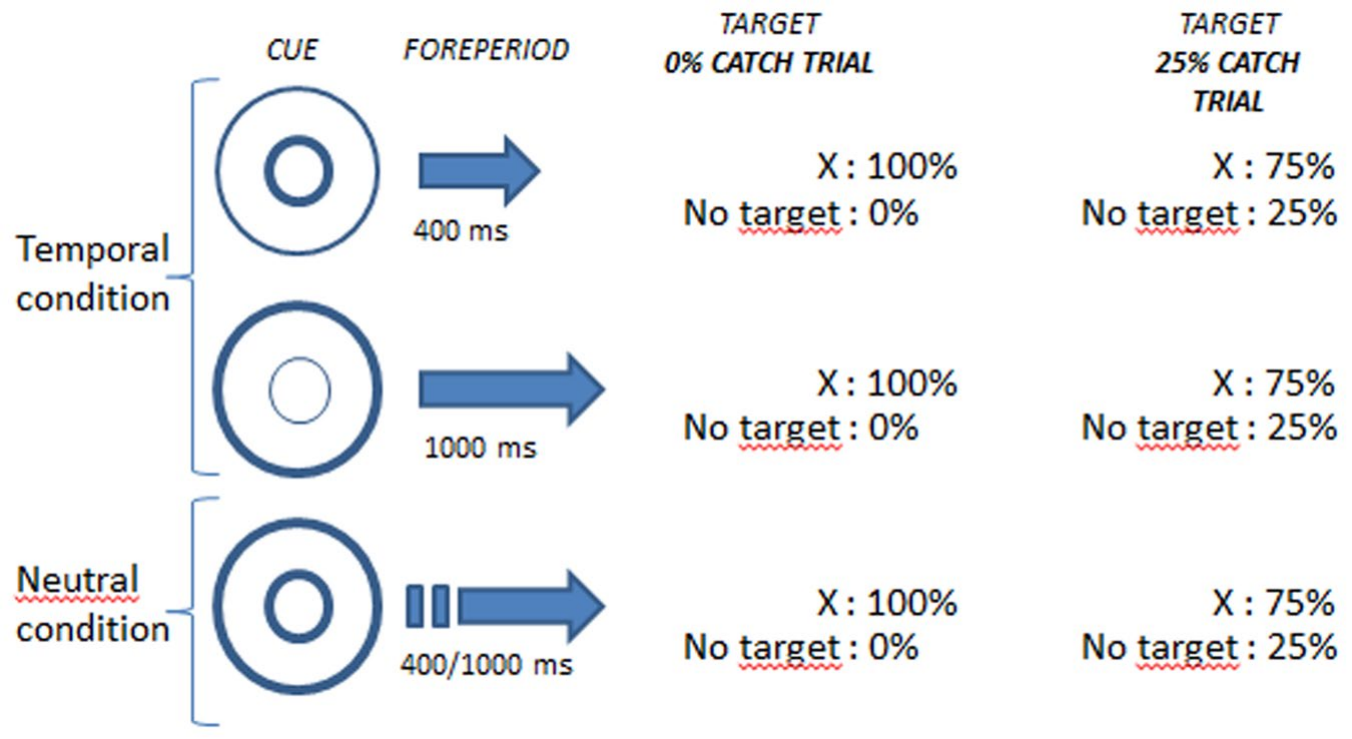
Experimental conditions. In the temporal condition, cues indicated that the target would appear after either a short (top row) or long (middle row) foreperiod. In the neutral condition (bottom row) cues provided no temporal information and the target was equally likely to appear after a short or long foreperiod. Targets were either present on every trial (“without catch trials” condition) or were absent on 25% of trials in the block (“with catch trials” condition).

In four of the blocks, the target was displayed in all trials: we refer to this condition as the ‘0% catch trials’ condition (Fig. 5). In the other four blocks, the target was absent in 25% of the trials. These trials are catch trials, and this condition is referred to as the ‘25% catch trials’ condition. All participants performed four consecutive blocks without catch trials (0% catch trial), for which there were two blocks with the temporal cue (T) and two blocks with the neutral cue (N), either in the order NTTN or TNNT. The four blocks with catch trials (25% catch trials) were presented in the same order. The order of blocks with, or without, catch trials was randomized across participants, as was the order of temporal versus neutral cue blocks.

### Statistical analysis

Trials with correct responses faster than 150 ms or slower that 1000 ms were excluded from the RT analysis (<3% trials in each group). Errors were rare (<2%) in both groups and so not analysed.

Mean RTs were submitted to an analysis of variance (ANOVA) with foreperiod (400 ms/1000 ms), cue type (temporal/neutral) and catch-trial percentage (0%/25%) as independant within-subjects variables and group (controls/patients) as a between-subject categorical variable. We decomposed interactions by means of Tukey post hoc analyses.

### Data availability statement

The datasets generated during and/or analysed during the current study are available from the corresponding author upon reasonable request.

## Electronic supplementary material

Complementary results
