# Supplementary material for: Fragile temporal prediction in patients with schizophrenia is related to minimal self disorders
Source: Sci Rep. 2017 Aug 15;7:8278. doi: 10.1038/s41598-017-07987-y (PMC5557764; doi:10.1038/s41598-017-07987-y)
Supplement: Supplementary file 1 — Complementary results [file 41598_2017_7987_MOESM1_ESM.pdf]

Fragile temporal prediction in patients with schizophrenia is related to minimal self disorders

Brice Martin[1], Nicolas Franck[1], Michel Cermolacce[3], Agnès Falco[1], Anabel Benair[1], Estelle Etienne[1], Sébastien Weibel[2], Jennifer T Coull [4], Anne Giersch[2]

[1] Centre Ressource de Réhabilitation psychosociale et de remédiation cognitive, Hôpital du Vinatier Centre référent Lyonnais en Réhabilitation et en Remédiation cognitive (CL3R), UMR 5229 (CNRS), France. brice.martin@ch-le-vinatier.fr

[2]INSERM U1114, Pôle de Psychiatrie, Fédération de Médecine Translationnelle de Strasbourg (FMTS), Centre Hospitalier Régional Universitaire of Strasbourg, Université de Strasbourg, France.

[3]Service universitaire de psychiatrie, Hôpital Ste Marguerite, 13000 Marseille

[4] Laboratoire des Neurosciences Cognitives (UMR 7291), Aix-Marseille Université & CNRS, 3 Place Victor Hugo, 13331 Marseille cedex 3

Supplementary material

Complementary results

Sub-group analysis on the basis of the EASE scores

We conducted a median split on the EASE self-awareness and presence scores and defined two groups based on a score of 5. Twelve patients had a score below 5, and 13 had a score equal to or higher than 5. An analysis of variance was conducted on mean RTs for neutral cue trials in the 0% catch trial condition, with foreperiod as a within-group variable and group as between-group variable (Figure S1). There was a significant interaction between foreperiod and group ($F[1, 22] = 4.7$, $p < 0.05$, partial $\eta^2=.18$). Post-hoc Tukey analyses showed that the group with high self-awareness and presence scores displayed no RT differences between the 400 ms and 1000 ms foreperiods, whereas the usual benefit of the hazard function on RTs at the 1000ms foreperiod was observed for the group with low self-awareness and presence scores ($p < 0.05$).

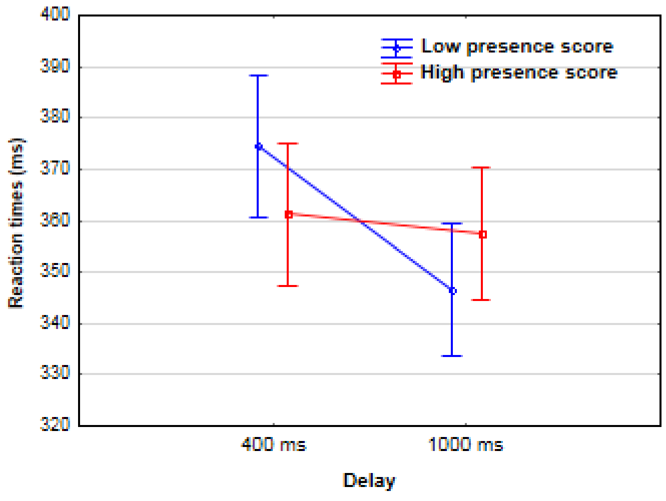

Figure S1: Response times (RT) in each patient group (those with low ‘self-awareness and presence’ scores in blue and those with high ‘self-awareness and presence’ scores in red) as a function of the foreperiod delay between the cue and the target (400 vs. 1000 ms). RT data were collected in the 100% target condition with neutral cues. A higher ‘self-awareness and presence’ score corresponds to more severe symptoms.

We additionally analyzed the influence of whether the preceding trial was a catch trial or not on RTs at 400 vs. 1000 ms. There was a significant interaction between foreperiod, group, and the presence vs. absence of a target in the preceding trial (F[1, 22] = 8.4, p < .01, partial η^2=.28). RTs increased, or tended to increase, from 400 to 1000 ms in patients with high self-awareness and presence scores, whether the preceding trial was a catch trial or not (by 21 ms after a catch trial, p < .005, and by 15 ms after a target-present trial, p = 0.07). By contrast, in patients with low self-awareness and presence scores, RTs increased only after a catch trial (by 32 ms, p < .001), but not after a target-present trial (RTs decreased by 5 ms, *n.s.*).

Complementary statistics

The entire set of results from the global ANOVA are detailed below:

	Mean RT in ms (SD)	Mean RT in ms (SD)	F	Df	P	Cohen's d	Effect-size r
Main effect of group							
	Patients 371.6 (38.6)	Controls 354.6 (53.3)	1.7	1,48	0.2	0.37	0.18
Main effect of catch-trial percentage							
	Without catch trials 351.4 (46.3)	With catch trials 376.2 (47.6)	119.4	1,48	0.000	0.53	0.26
Effect of catch-trial percentage x group							
			0.2	1,48	0.6		
Main effect of foreperiod							
	400 ms 365.4 (50)	1000 ms 362.1 (44.8)	1.22	1,48	0.27	0.07	0.03
Foreperiod x group							
			0.14	1,48	0.7		
Main effect of cue type							
	With neutral cues 366.6 (46.6)	With temporal cues 361 (46.4)	13.9	1,48	0.0005	0.12	0.06
Cue type x group							
			0.007	1,48	0.9		
Effect of catch-trial percentage x foreperiod							
			62.4	1,48	0.000		

Effect of catch-trial percentage x foreperiod x group							
			4.5	1, 48	0.039		
Effect of catch-trial percentage x cue type							
			0.5	1, 48	0.5		
Effect of catch-trial percentage x cue type x group							
			0.1	1, 48	0.5		
Effect of foreperiod x cue type							
			10.6	1, 48	0.002		
Effect of foreperiod x cue type x group							
			0.9	1, 48	0.4		
Effect of catch-trial percentage x foreperiod x cue type							
			3.6	1, 48	0.06		
Effect of catch-trial percentage x foreperiod x cue type x group							
			0.4	1, 48	0.6		

Table S1: Detailed statistics from the global ANOVA

Bayesian analysis

In order to verify the increase in RT between the 400 and 1000 ms foreperiods in the catch trial block, we conducted a Bayesian analysis of variance with repeated measures, using a backward stepwise analysis from the complete model with all interactions. MCMC chains were conducted with R and JAGS, with 100 000 iterations performed after 5000 burn-in iterations. Uninformed priors were used. Contrasts of interest were calculated from the selected model, i.e. mean RT difference between trials with a foreperiod of 400 and 1000ms, in the block with 25% catch trials, for each group (controls and patients).

In the control group, the difference in RT was estimated to be 0.55 ms (95% credibility interval [-4.57; 5.64]). The probability of a difference>0 was estimated to be 54.8%. In the patient group, the RT difference was 18.15 ms (95% credibility interval [-5.32; 41.54]), and the probability of a difference>0 was estimated to be 93.8% (see Figure S2 for the magnitude of the RT slope between the 400 and 1000 ms foreperiods, in the 0% and 25% catch trials conditions, in individual participants).

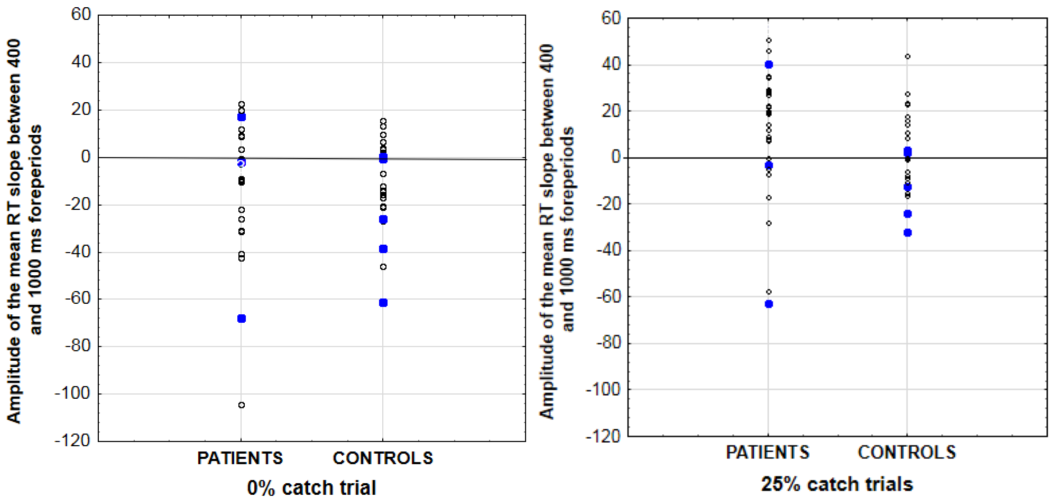

Figure S2 : Individual participant performance in terms of the magnitude of the RT slope between the 400 and 1000 ms foreperiods, in the 0% (left panel) and 25% (right panel) catch trial conditions, in either patients (on the left of the graphs) or controls (on the right of the graphs). Positive slopes (above the 0 line) represent an increase in RT from 400 to 1000 ms, whereas negative slopes (below the 0 line) represent a decrease.

Correlations

We report below the entire set of correlations between different EASE sub-scale scores and (1) the magnitude of the slope between short and long foreperiods in the 0% catch trial condition, (2) the magnitude of the same slope in the 25% catch trial condition, and (3) the change in RT slope for trials that followed a catch trial vs. those that followed a target-present trial.

	EASE Total	EASE Cognition and stream of consciousness	EASE Self awareness and presence	EASE Bodily experiences	EASE Demarcation transitivism	EASE Existential reorientation
**0% catch trials** slope between short and long foreperiods (neutral cue)	-.2992 p=.165	-.1154 p=.600	-.4218 p=.045	-.1782 p=.416	-.2302 p=.291	-.2607 p=.229
**25% catch trials** slope between short and long foreperiods (neutral cue)	-.1359 p=.537	.0064 p=.977	-.1645 p=.453	-.1338 p=.543	-.0660 p=.765	-.4395 p=.036
change in RT slope after a catch trial	-.3011 p=.163	-.1379 p=.530	-.5771 p=.004	-.2874 p=.184	-.1680 p=.443	-.0301 p=.892

Table S2: Correlation values (r) and associated statistical significance between EASE sub-scale scores and the benefits of the hazard function on performance (i.e. the magnitude of the slope between short and long foreperiods) in the 0% or 25% catch trial condition, or between EASE scores and the effect of a recent catch trial on performance (i.e. the change in RT slope for trials that followed a catch trial vs. those that followed a target-present trial).
